# Picking a bone with WISP1 (CCN4): new strategies against degenerative joint disease

**Published:** 2016-01-26

**Authors:** Kenneth Maiese

**Affiliations:** Cellular and Molecular Signaling, Newark, New Jersey 07101, USA

**Keywords:** Akt, apoptosis, arthritis, autophagy, degenerative joint disease, IL-6, microRNA, mTOR, *NF*-κ*B*, osteoarthritis, PI 3-K, SIRT1, stem cells, WISP1, Wnt signaling

## Abstract

As the world’s population continues to age, it is estimated that degenerative joint disease disorders such as osteoarthritis will impact at least 130 million individuals throughout the globe by the year 2050. Advanced age, obesity, genetics, gender, bone density, trauma, and a poor level of physical activity can lead to the onset and progression of osteoarthritis. However, factors that lead to degenerative joint disease and involve gender, genetics, epigenetic mechanisms, and advanced age are not within the control of an individual. Furthermore, current therapies including pain management, improved nutrition, and regular programs for exercise do not lead to the resolution of osteoarthritis. As a result, new avenues for targeting the treatment of osteoarthritis are desperately needed. Wnt1 inducible signaling pathway protein 1 (WISP1), a matricellular protein and a downstream target of the wingless pathway Wnt1, is one such target to consider that governs cellular protection, stem cell proliferation, and tissue regeneration in a number of disorders including bone degeneration. However, increased WISP1 expression also has been associated with the progression of osteoarthritis. WISP1 has an intricate relationship with a number of proliferative and protective pathways that include phosphoinositide 3-kinase (PI 3-K), protein kinase B (Akt), nuclear factor kappa-light-chain-enhancer of activated B cells (NF-κB), interleukin -6 (IL-6), transforming growth factor-β, matrix metalloproteinase, small non-coding ribonucleic acids (RNAs), sirtuin silent mating type information regulation 2 homolog 1 (Saccharomyces cerevisiae) (SIRT1), and the mechanistic target of rapamycin (mTOR). Taken together, this complex association WISP1 holds with these signaling pathways necessitates a fine biological regulation of WISP1 activity that can offset the progression of degenerative joint disease, but not limit the cellular protective capabilities of the WISP1 pathway.

## The fine targeting of WISP1 for degenerative joint disease

Osteoarthritis is a chronic disorder that results from cartilage degeneration and mechanical stress imposed upon the skeletal system. Low bone mass and structural deterioration of bone tissue eventually lead to bone fragility and increased susceptibility to fractures. As a result, individuals with this disorder have impaired mobility and pain that can occur in hip joints, the shoulder, spine, knees, feet, and hands. The most severe fracture that can result from osteoarthritis involves the hip that requires hospitalization and leads to permanent disability in 50% of individuals and fatality in another 20% of individuals.

In developed nations, osteoarthritis is considered to be one of the ten most common disabilities in aged individuals especially those that remain active in the workforce [1]. According to the World Health Organization [2], at least 15% of all adults over the age of 60 are believed to suffer from this disorder with women having greater prevalence of osteoarthritis than men. It is estimated that worldwide 9.6% of men and 18.0% of women over the age of 60 suffer with osteoarthritis. With advancing age of the world’s population, the incidence of osteoarthritis is expected to increase. By the year 2050, at least 130 million people throughout the world may suffer from osteoarthritis.

Risk factors that can lead to the progression of osteoarthritis involve advanced age, obesity, genetics, gender, bone density, trauma, and a poor level of physical activity [3]. Preventive measures can be instituted to decrease the onset of osteoarthritis such as protective clothing and gait aides to avoid trauma, regular programs for exercise, and nutritional programs that address proper weight management. Yet, factors such as gender, genetics, and advanced age are beyond an individual’s control and therapies directed at pain management offer symptomatic relief at best. Furthermore, complex epigenetic mechanisms that oversee DNA methylation, small non-coding RNAs (microRNAs), post-translation protein modification, and histone deacetylation may present additional risk factors for the development of osteoarthritis.

One exciting therapeutic target for osteoarthritis that is emerging as a novel consideration is Wnt1 inducible signaling pathway protein 1 (WISP1), also known as CCN4 [4-6]. WISP1 is a matricellular protein and a downstream target of the *wingless* pathway Wnt1 [7]. In addition, WISP1 is a member of the CCN family of proteins. The CCN family of proteins contains six secreted extracellular matrix associated proteins. They are defined by the first three members of the family that include Cysteine-rich protein 61, Connective tissue growth factor, and Nephroblastoma over-expressed gene [8,9]. WISP1 is expressed in the brain, heart, kidney, lung, pancreas, placenta, epithelium, ovaries, small intestine, and spleen [9]. Of interest, WISP1 can govern cellular survival, metabolism, and stem cell proliferation and maintenance [10] and can modulate epigenetic pathways [9-11].

WISP1 may be important for tissue repair and regeneration during a number of diseases. For example, WISP1 can control induced pluripotent stem cell reprogramming [12,13] and is one of several genes that are over-expressed during pancreatic regeneration [14]. WISP1 also can foster vascular regeneration during saphenous vein crush injury [15]. WISP1 expression is increased during stem cell migration [16] and is repressed during hepatic differentiation in adipose-derived stem cells [17]. WISP1 leads to vascular smooth muscle proliferation that can assist with tissue repair during injury [18,19]. WISP1 also is tightly linked to metabolic homeostasis [14] and appears to have a modulatory role in cell senescence. WISP1 can control cellular senescence [20] to a degree that does not promote excessive cellular proliferation in aging vascular cells [21] that could lead to atherosclerosis during diabetes mellitus.

In regards to the musculoskeletal system, WISP1 has been shown to promote mesenchymal cell proliferation and osteoblastic differentiation with the repression of chondrocytic differentiation to further bone development [22] and assist with fracture repair [23]. Bone formation after growth plate cartilage injury involves expression of the *WISP1* gene [24]. WISP1 may increase osteogenesis activity through bone morphogenetic protein 2 [25] and be required for bone formation through parathyroid hormone treatment [26]. WISP1 also oversees bone morphogenetic protein-3 stimulated mesenchymal stem cell proliferation [27].

Given the ability of WISP1 to control cellular proliferation in the musculoskeletal system, WISP1 and related members of the CCN family have emerged as potential targets for disorders such as osteoarthritis and rheumatoid arthritis. CCN1, CCN2, CCN4, and CCN5 have been found to be expressed to a greater extent in knee cartilage during osteoarthritis and rheumatoid arthritis when compared to normal controls [28]. In particular, WISP1 is considered a significant factor for the progression of osteoarthritis. In osteoarthritis synovial fibroblasts, WISP1 can activate αvβ5 integrin, phosphoinositide 3-kinase (PI 3-K), protein kinase B (Akt), and nuclear factor kappa-light-chain-enhancer of activated B cells (NF-κB) pathways that result in the up-regulation of interleukin -6 (IL-6) production [29]. WISP1 leads to chondrocyte hypertrophy through transforming growth factor-β (TGF-β) signaling and activin-like kinase (ALK)1/Smad 1/5/8 pathway [30]. In models of osteoarthritis, WISP1 controls chondrocyte and macrophage matrix metalloproteinase and aggrecanase expression that results in articular cartilage damage [31]. Furthermore, overexpression of WISP1 can lead to increased cartilage damage while blocking of the upstream canonical Wnt signaling pathway can limit cartilage damage [32]. Interestingly, the detrimental effects of WISP1 in arthritic disease somewhat parallel the ability of WISP1 to also promote fibrotic tissue injury. WISP1 expression has been tied to idiopathic pulmonary fibrosis possibly regulated by the microRNA miR-92a [33] and linked to fibrosis in models of liver fibrogenesis [34].

In light of the emerging knowledge of WISP1 signaling pathways, promoting the down-regulation of WISP1 expression in arthritic joint disease appears to open new therapeutic strategies for this disabling disorder. Yet, WISP1 also is associated with a number of cellular pathways that are supportive of bone development and repair and protective against cell injury that include silent mating type information regulation 2 homolog 1 *(Saccharomyces cerevisiae*) SIRT1 [35] and mechanistic target of rapamycin (mTOR) (36). WISP1 can protect cells against oxidative stress [35,37], control pathways of apoptosis and autophagy [36,38], and prevent inflammatory cell injury during exposure to toxins such as β-amyloid [39,40]. Therefore, a careful targeted approach that limits WISP1 activity but does not negatively impact cellular protective pathways may be required for the development of WISP1 as a novel treatment for musculoskeletal disorders.

## References

[1] Palmer KT, Goodson N (2015). Ageing, musculoskeletal health and work. Best Pract Res Clin Rheumatol.

[2] WHO Department of Chronic Diseases and Health Promotion http://www.who.int/chp/topics/rheumatic/en/.

[3] Gabay O, Clouse KA (2015). Epigenetics of cartilage diseases. Joint Bone Spine.

[4] Maeda A, Ono M, Holmbeck K, Li L, Kilts TM (2015). WNT1-induced Secreted Protein- ( WISP1), a Novel Regulator of Bone Turnover and Wnt Signaling. J Biol Chem.

[5] Maiese K (2015). Novel applications of trophic factors, Wnt and WISP for neuronal repair and regeneration in metabolic disease. Neural Regen Res.

[6] Murahovschi V, Pivovarova O, Ilkavets I, Dmitrieva RM, Döcke S (2015). WISP1 is a novel adipokine linked to inflammation in obesity. Diabetes.

[7] Maiese K, Li F, Chong ZZ, Shang YC (2008). The Wnt signaling pathway: aging gracefully as a protectionist?. Pharmacol Ther.

[8] Krupska I, Bruford EA, Chaqour B (2015). Eyeing the Cyr61/CTGF/NOV (CCN) group of genes in development and diseases: highlights of their structural likenesses and functional dissimilarities. Hum Genomics.

[9] Maiese K (2014). WISP1: Clinical insights for a proliferative and restorative member of the CCN family. Curr Neurovasc Res.

[10] Maiese K (2015). New Insights for Oxidative Stress and Diabetes Mellitus. Oxid Med Cell Longev.

[11] Maiese K (2015). FoxO Transcription Factors and Regenerative Pathways in Diabetes Mellitus. Curr Neurovasc Res.

[12] Jung DW, Kim WH, Williams DR (2014). Reprogram or reboot: small molecule approaches for the production of induced pluripotent stem cells and direct cell reprogramming. ACS Chem Biol.

[13] Yang CS, Lopez CG, Rana TM (2011). Discovery of nonsteroidal anti-inflammatory drug and anticancer drug enhancing reprogramming and induced pluripotent stem cell generation. Stem Cells.

[14] Lim HW, Lee JE, Shin SJ, Lee YE, Oh SH (2002). Identification of differentially expressed mRNA during pancreas regeneration of rat by mRNA differential display. Biochem Biophys Res Commun.

[15] Price RM, Tulsyan N, Dermody JJ, Schwalb M, Soteropoulos P (2004). Gene expression after crush injury of human saphenous vein: using microarrays to define the transcriptional profile. J Am Coll Surg.

[16] Lough D, Dai H, Yang M, Reichensperger J, Cox L (2013). Stimulation of the follicular bulge LGR5+ and LGR6+ stem cells with the gut-derived human alpha defensin 5 results in decreased bacterial presence, enhanced wound healing, and hair growth from tissues devoid of adnexal structures. Plast Reconstr Surg.

[17] Heo J, Ahn EK, Jeong HG, Kim YH, Leem SH (2013). Transcriptional characterization of Wnt pathway during sequential hepatic differentiation of human embryonic stem cells and adipose tissue-derived stem cells. Biochem Biophys Res Commun.

[18] Liu H, Dong W, Lin Z, Lu J, Wan H (2013). CCN4 regulates vascular smooth muscle cell migration and proliferation. Mol Cells.

[19] Reddy VS, Valente AJ, Delafontaine P, Chandrasekar B (2011). Interleukin-18/WNT1-inducible signaling pathway protein-1 signaling mediates human saphenous vein smooth muscle cell proliferation. J Cell Physiol.

[20] Du J, Klein JD, Hassounah F, Zhang J, Zhang C (2014). Aging increases CCN1 expression leading to muscle senescence. Am J Physiol Cell Physiol.

[21] Marchand A, Atassi F, Gaaya A, Leprince P, Le Feuvre C (2011). The Wnt/beta-catenin pathway is activated during advanced arterial aging in humans. Aging Cell.

[22] Thorfve A, Lindahl C, Xia W, Igawa K, Lindahl A4 (2014). Hydroxyapatite coating affects the Wnt signaling pathway during peri-implant healing in vivo. Acta Biomater.

[23] French DM, Kaul RJ, D'Souza AL, Crowley CW, Bao M (2004). WISP-1 is an osteoblastic regulator expressed during skeletal development and fracture repair. Am J Pathol.

[24] Macsai CE, Georgiou KR, Foster BK, Zannettino AC, Xian CJ (2012). Microarray expression analysis of genes and pathways involved in growth plate cartilage injury responses and bony repair. Bone.

[25] Ono M, Inkson CA, Kilts TM, Young MF (2011). WISP-1/CCN4 regulates osteogenesis by enhancing BMP-2 activity. J Bone Miner Res.

[26] Saidak Z, Le Henaff C, Azzi S, Marty C, Marie PJ (2014). Low-dose PTH increases osteoblast activity via decreased Mef2c/Sost in senescent osteopenic mice. J Endocrinol.

[27] Cernea M, Tang W, Guan H, Yang K (2016). Wisp1 mediates Bmp3-stimulated mesenchymal stem cell proliferation. J Mol Endocrinol.

[28] Komatsu M, Nakamura Y, Maruyama M, Abe K, Watanapokasin R (2015). Expression profiles of human CCN genes in patients with osteoarthritis or rheumatoid arthritis. J Orthop Sci.

[29] Hou CH, Tang CH, Hsu CJ, Hou SM, Liu JF (2013). CCN4 induces IL-6 production through alphavbeta5 receptor, PI3K, Akt, and NF-kappaB singling pathway in human synovial fibroblasts. Arthritis Res Ther.

[30] van den Bosch MH, Blom AB, van Lent PL, van Beuningen HM4, Blaney Davidson EN5 (2014). Canonical Wnt signaling skews TGF-Î^2^ signaling in chondrocytes towards signaling via ALK1 and Smad 1/5/8. Cell Signal.

[31] Blom AB, Brockbank SM, van Lent PL, van Beuningen HM, Geurts J (2009). Involvement of the Wnt signaling pathway in experimental and human osteoarthritis: prominent role of Wnt-induced signaling protein 1. Arthritis Rheum.

[32] van den Bosch MH, Blom AB, Sloetjes AW, Koenders MI, van de Loo FA (2015). Induction of Canonical Wnt Signaling by Synovial Overexpression of Selected Wnts Leads to Protease Activity and Early Osteoarthritis-Like Cartilage Damage. Am J Pathol.

[33] Berschneider B, Ellwanger DC, Baarsma HA, Thiel C, Shimbori C (2014). miR-92a regulates TGF-Î^2^1-induced WISP1 expression in pulmonary fibrosis. Int J Biochem Cell Biol.

[34] Jian YC, Wang JJ, Dong S, Hu JW, Hu LJ (2014). Wnt-induced secreted protein 1/CCN4 in liver fibrosis both in vitro and in vivo. Clin Lab.

[35] Wang S, Chong ZZ, Shang YC, Maiese K (2013). WISP1 neuroprotection requires FoxO3a post-translational modulation with autoregulatory control of SIRT1. Curr Neurovasc Res.

[36] Shang YC, Chong ZZ, Wang S, Maiese K (2013). Tuberous sclerosis protein ( TSC2) modulates CCN4 cytoprotection during apoptotic amyloid toxicity in microglia. Curr Neurovasc Res.

[37] Wang S, Chong ZZ, Shang YC, Maiese K (2012). Wnt1 inducible signaling pathway protein ( WISP1) blocks neurodegeneration through phosphoinositide 3 kinase/Akt1 and apoptotic mitochondrial signaling involving Bad, Bax, Bim, and Bcl-xL. Curr Neurovasc Res.

[38] Wang S, Chong ZZ, Shang YC, Maiese K (2012). WISP ( CCN4) autoregulates its expression and nuclear trafficking of Î^2^-catenin during oxidant stress with limited effects upon neuronal autophagy. Curr Neurovasc Res.

[39] Maiese K (2015). Targeting molecules to medicine with mTOR, autophagy, and neurodegenerative disorders. Br J Clin Pharmacol.

[40] Shang YC, Chong ZZ, Wang S, Maiese K (2012). Wnt1 inducible signaling pathway protein ( WISP1) targets PRAS40 to govern Î^2^-amyloid apoptotic injury of microglia. Curr Neurovasc Res.

